# Giant Poroid Hidradenoma of the Forearm

**DOI:** 10.7759/cureus.52047

**Published:** 2024-01-10

**Authors:** Nadir Miry, Anass Haloui, El Mehdi Tiabi, Nassira Karich, Amal Bennani

**Affiliations:** 1 Pathology Department, Faculty of Medicine and Pharmacy, Mohammed VI University Hospital, Oujda, MAR

**Keywords:** sweat gland tumor, skin adnexal tumor, poroid neoplasm, poroid hidradenoma, hidradenoma

## Abstract

Poroid hidradenoma represents an uncommon and benign tumor originating from skin adnexa. It falls under the category of sweet duct neoplasms, along with poromas. It affects the elderly population most frequently. Typically, it emerges as a small, distinct, and painless lump beneath the skin's surface, often occurring on the head and neck regions. It is characterized by a low risk of malignant transformation. Accurate identification relies especially on histomorphological analysis considering the intricate resemblance it shares with other tumors originating from eccrine glands. Poroid hidradenoma has only recently been recognized, and only a limited number of cases have been reported in the medical literature. In this instance, we present an unusual occurrence of a giant poroid hidradenoma on the left forearm of an elderly patient.

## Introduction

Poroid hidradenoma is a rare and benign cutaneous neoplasm, originating from eccrine glands, which presents as a solitary lesion frequently located in the head and neck regions. Initially described by Abenoza and Ackerman, it predominantly affects older patients [1]. It is an adnexal tumor with eccrine differentiation, associated with a very low risk of malignant transformation [2]. It falls within the category of poroid neoplasms, which includes classic poroma, dermal duct tumor, hidroacanthoma simplex, and, finally, poroid hidradenoma. Its management is based on a complete resection of the lesion to reduce the risk of recurrence [1].

We describe here the case of a 74-year-old patient who presented with a giant mass on his left forearm that had persisted over four years, and the lesion was histologically consistent with a poroid hidradenoma.

## Case presentation

**Figure 1:**
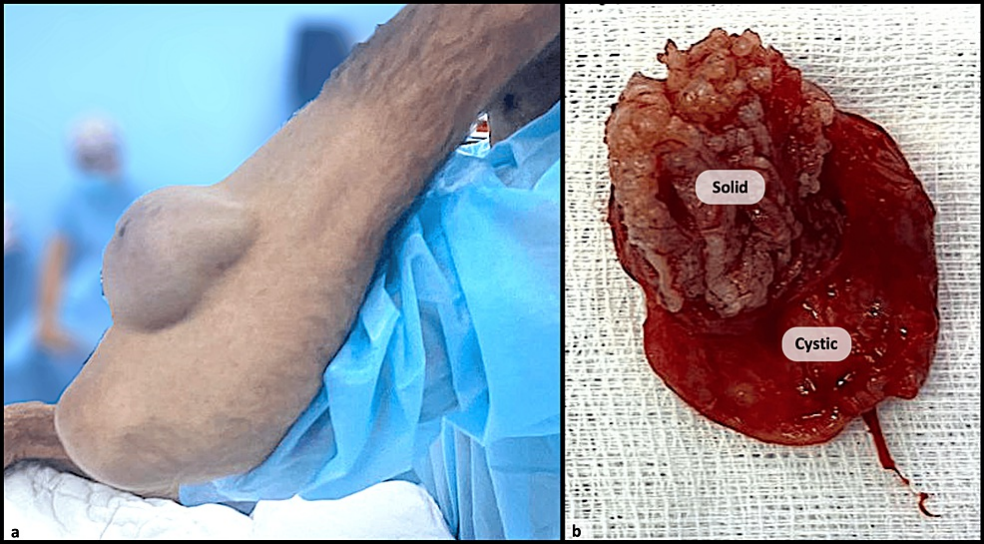
Mass lesion of the forearm (a). Macroscopically, the lesion is composed of solid and cystic components (b).

We report here the case of a 74-year-old male who presented to the orthopedic department with a large mass in the posterior compartment of his left forearm. The mass had a slow-growing character over four years. The patient did not report any pain, color changes of the overlying skin, or any discharge. Additionally, no general symptoms, such as fever or weight loss, were reported. However, he did experience mild discomfort during his daily activities because of the mass's increasing size. Upon cutaneous examination, the lesion was solitary, non-tender, and firm, measuring 10 cm with well-defined borders, and no signs of ulceration or surface changes (Figure 1a). No other abnormalities were noted during the rest of the physical examination. A subcutaneous dissection of the mass from the surrounding tissue was performed.

Macroscopically, the specimen measured 8x5x1cm and exhibited a thin-walled cystic structure containing a solid region with an irregular surface (Figure 1b).

**Figure 2:**
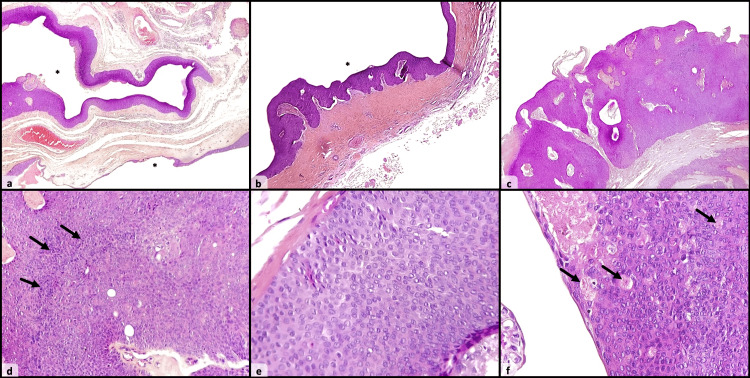
Photomicrographs of the lesion showing a predominantly cystic mass, with a central lumen (*), and no surrounding capsule (a: HES, x40, and b: HES, x100). The lesion shows both cystic and solid components (c: HES: x100). Solid areas are composed of two types of cells: dark poroid cells (arrows) and eosinophilic cuticular cells (d: HES, x200). At higher magnification, cuticular cells show abundant eosinophilic cytoplasm with a large central nucleus (e: HES, x400). Multiple ducts were seen (arrows), reminiscent of ductal differentiation (f: HES, x400). HES: hematoxylin-eosin-saffron staining.

The nature of the cyst's content could not be definitively determined as it had been inadvertently cut open during the surgical procedure. The histopathological examination revealed an epithelial tumor, located within the dermis and extending into the subcutaneous region. The tumor was unencapsulated and lacked any connection to the epidermal skin layer. It combined architectural elements resembling those of hidradenoma, including both solid and cystic areas (Figures 2a, 2b, 2c), as well as cytological characteristics of poroma. The latter consisted of two cell types: poroid cells, characterized by smaller size and dark-stained nuclei; and cuticular cells, which are larger with an abundant eosinophilic cytoplasm. Both cell types displayed a uniform appearance (Figures 2d, 2e). Some areas showed signs of degeneration marked by cystic changes. Some regions within the tumor exhibited duct-like structures (Figure 2f).

The possibility of a poroma was unlikely since there was no connection to the epidermis or evident cord-like structures. Hidradenoma simplex was ruled out due to its defining criteria which makes it an entirely intraepidermal lesion. The absence of a nested arrangement and epidermal connection reduced the likelihood of a dermal ductal tumor. The possibility of a hidradenocarcinoma was also minimal as there were no atypical cells or mitotic figures throughout the lesion. The diagnosis of a poroid hidradenoma was established based on these findings. Following six months of follow-up, the patient did not experience any recurrence or complications linked to the surgery.

## Discussion

Poroid hidradenoma is an uncommon tumor arising from adnexal structures, displaying eccrine differentiation. It falls under the category of poroid neoplasms, accounting for less than 5% of all hidradenomas [2]. The hidradenoma category includes both poroid and apocrine types, showing close histomorphological similarities that can pose challenges in differentiation.

Poroid hidradenoma is more frequently diagnosed in the elderly population, showing no apparent gender predilection [3]. Clinically, it presents as a solitary nodule located within the dermal layer of the skin. Typically, painless, small in size, and measures between one and two centimeters in its largest diameter [4]. Interestingly, our patient exhibited an unusually large PH, which is atypical for such benign adnexal tumors. These neoplasms are commonly located on the head and neck regions, and less frequently on the trunk and extremities [3]. Grossly, PH comprises both solid and cystic components.

Histologically, it appears as a well-circumscribed lesion confined to the dermis, featuring both solid and cystic areas. Solid areas consist of two types of cells: poroid cells, which are small and cuboidal with round nuclei, and cuticular cells, larger and polygonal, with relatively abundant eosinophilic cytoplasm and a large, pale nucleus. Different types of cells are usually distributed randomly throughout the tumor or concentrated in clusters within the tumor lobules. The cystic component is often large and contains amorphous eosinophilic secretions, reminiscent of the eccrine nature of the tumor [5,6]. Necrosis is frequently observed, appearing strongly eosinophilic at the center of tumor lobules.

Immunohistochemical analysis serves as an ancillary tool, highlighting ductal differentiation throughout EMA positivity [7], and ki67 demonstrates a low proliferative index. However, in our case, immunohistochemical markers were unnecessary, as the histomorphological characteristics were sufficient to confirm the diagnosis and rule out potential differentials. Otherwise, hidroacanthoma simplex is characterized by nests of round cells located in the epidermis [6]. Dermal duct tumor is located within the dermis and consists of multiple well-demarcated small nests, without any connections to the epidermis. Eccrine poroma is usually a well-circumscribed tumor displaying a lobular pattern, with epidermal connection as a distinctive feature [8]. Finally, apocrine hidradenomas are recognized by their characteristic decapitation secretions.

Even though the risk of malignant transformation is very low, it is recommended to perform a complete excision to remove the skin and surrounding soft tissue for such lesions to prevent any recurrence. A follow-up period is also recommended [9].

## Conclusions

Poroid hidradenoma is a rare and benign adnexal tumor belonging to poroid neoplasms, it is characterized by a very low risk of malignant transformation. While only a few cases have been documented to date, more studies are needed to fully understand the behavior of such tumors, particularly their risk of recurrence and malignant transformation. In this case report, we highlight the unusual presentation of a giant poroid hidradenoma that evolved over four years without showing any signs of malignant transformation.

## References

[1] Reddy R, Apoorva H, Eshwari L (2022). Poroid hidradenoma over dorsum of the hand. Clin Dermatol Rev.

[2] Sruthi S (2020). A rare case of poroid hidradenoma scalp. Univ J Surg Surg Spec.

[3] Lim JS, Kwon ES, Myung KB, Cheong SH (2021). Poroid hidradenoma: a two-case report and literature review. Ann Dermatol.

[4] Roodsari MR, Abdolghafoorian H, Saiedi M (2013). Poroid hidradenoma: a rare tumor entity. J Case Rep.

[5] Mukit M, Mitchell M, Ortanca I, Krassilnik N, Jing X (2021). Poroid hidradenoma of the scalp in a US Veteran's Administration (VA) patient. Case Reports Plast Surg Hand Surg.

[6] Battistella M, Langbein L, Peltre B, Cribier B (2010). From hidroacanthoma simplex to poroid hidradenoma: clinicopathologic and immunohistochemic study of poroid neoplasms and reappraisal of their histogenesis. Am J Dermatopathol.

[7] Requena L, Sánchez M (1992). Poroid hidradenoma: a light microscopic and immunohistochemical study. Cutis.

[8] Kumar P, Das A, Savant SS (2017). Poroid hidradenoma: an uncommon cutaneous adnexal neoplasm. Indian J Dermatol.

[9] Miller R, Ieremia E, Birch J, Chan J (2018). Poroid hidradenoma in the hand: a case report and systematic review. J Cutan Pathol.

